# Frictional melting mechanisms of rocks during earthquake fault slip

**DOI:** 10.1038/s41598-023-39752-9

**Published:** 2023-08-02

**Authors:** Sangwoo Woo, Raehee Han, Kiyokazu Oohashi

**Affiliations:** 1 Department of Geology and Research Institute of Natural Science, Gyeongsang National University, Jinju, 52828 Republic of Korea; 2 Graduate School of Sciences and Technology for Innovation, Yamaguchi University, Yamaguchi, 753-8512 Japan

**Keywords:** Geology, Geophysics

## Abstract

Rapid slip, at rates in the order of 1 m/s or more, may induce frictional melting in rocks during earthquakes. The short-lived melting has been thought to be a disequilibrium process, for decades. We conducted frictional melting experiments on acidic, basic, and ultrabasic silicate rocks at a slip rate of 1.3 m/s. The experiments and microstructural observations reveal that all minerals in the rocks are melted at temperatures below their known melting temperatures (T_m_); e.g., quartz is melted at ~ 1000–1200 °C, not ~ 1720 °C, while olivine at ~ 1300 °C, rather than ~ 1700 °C. The low-temperature melting is incompatible with the conventional disequilibrium melting, and may be caused predominantly by grain size reduction and phase boundary reactions during the early and later stages of slip, respectively. The newly estimated T_m_ and the melting mechanisms should be considered for understanding the mechanics of earthquakes, landslides, and caldera collapses.

## Introduction

Frictional melting is the extreme process resulting from shear heating caused by rapid slip in rock bodies. The rheology of rock melts in slip zones may control the mechanics of sliding—that is, lubricating or braking the slip—during dynamic events in nature, such as earthquakes^1^, landslides^2,3^, caldera collapses^4–6^, or meteorite impacts^7^. The question of how much temperature rise is necessary for frictional melting to occur is essential. Although it is a critical property for understanding frictional melting, rock melting temperatures during frictional sliding have rarely been estimated directly through experimentation^5,8–13^.

Frictional melting has mostly been discussed in terms of the melting temperatures (T_m_) of individual rock-forming minerals, as determined by static heating experiments (e.g., Table 1 in ref.^14^). It remains unclear whether such T_m_ are applicable to dynamic events, as additional slip zone processes—such as grain size reduction to ultrafine- or nano-sized particles, and phase transformation (e.g., the α–β transition of quartz, and amorphization)—may affect melting processes (e.g.,^14–19^). As frictional melting is a short-lived process (mostly < 10 s, except for large landslides and caldera collapses), it has been thought to be a disequilibrium melting, for several decades, despite the fact that the T_m_ of rock-forming minerals during rapid slip have not been determined experimentally^14,18,20–24^. The conventional view has been that reactions between minerals are insignificant, and the minerals having lower T_m_ could be selectively melted, while the minerals with higher T_m_ could survive the frictional heating^14,18^. It is only recently that a new perspective —that silica can be melted at lower temperatures than expected (~ 1350–1500 °C, instead of 1726 °C), and the frictional melting may be described by a combination of equilibrium thermodynamics and melting of metastable mineral phases (quasi-equilibrium melting)—has been proposed, based on a study of quartzite^15^.

In this study, we estimated the T_m_ for rock-forming minerals in common igneous rocks (from acidic to ultrabasic), and examined the melting mechanisms. This was achieved by integrating temperature measurements established during high-velocity frictional sliding tests, microstructural observation, and chemical analysis. Our results indicate that T_m_ during seismic slip can be significantly lower than previously thought, and the low-temperature melting may be caused by significant grain size reduction and phase boundary reactions.

## Results

### Frictional melting experiments and temperature measurements

**Figure 1:**
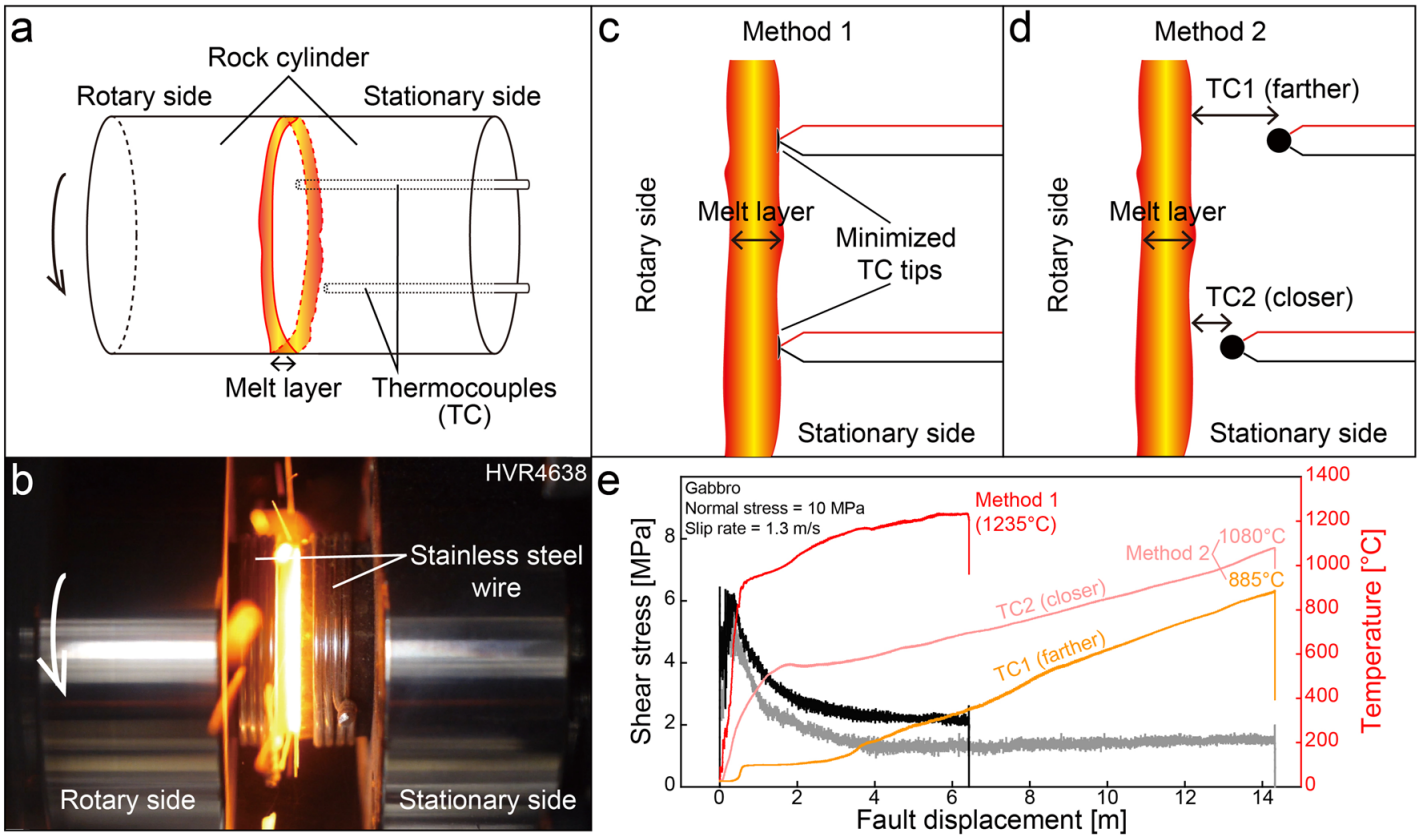
Frictional melting experiments and temperature estimation. (**a**) Specimen configuration. (**b**) Experimental photograph. (**c**) Method 1 for estimating melt layer boundary temperature (T_b_) based on a direct measurement by thermocouples (TC) initially set nearly at the same level as the fault surface. (**d**) Method 2 for estimating T_b_ using TC temperatures at different distances from the melt layer and measured axial displacement (or shortening) rate. The methods are described in the text and Methods. (**e**) Frictional behavior and temperature in experiments on gabbro.

Eighteen high-velocity rotary shear tests were conducted, at room temperature and room humidity, on solid cylindrical specimens of four igneous rocks, ranging from acidic to ultrabasic: granodiorite, gabbro, anorthosite, and peridotite (Fig. 1a, b; see Starting materials in “Methods”). The major minerals of the rocks were found to be albite and quartz in the granodiorite, labradorite (An_61_) and pigeonite in the gabbro, labradorite (An_61_) in the anorthosite, and olivine (Fo_84_), enstatite, and diopside in the peridotite (Fig. S1 and Table S1). The experimental normal stresses and the slip rate were 10–15 MPa and 1.3 m/s, respectively (Table S2). In each experiment, the shear stress increased to a peak, then decreased, and then increased again to another peak, before finally decreasing markedly to a steady-state value (Fig. 1e; see also Fig. S2 for the mechanical data related to all the rocks).

**Figure 2:**
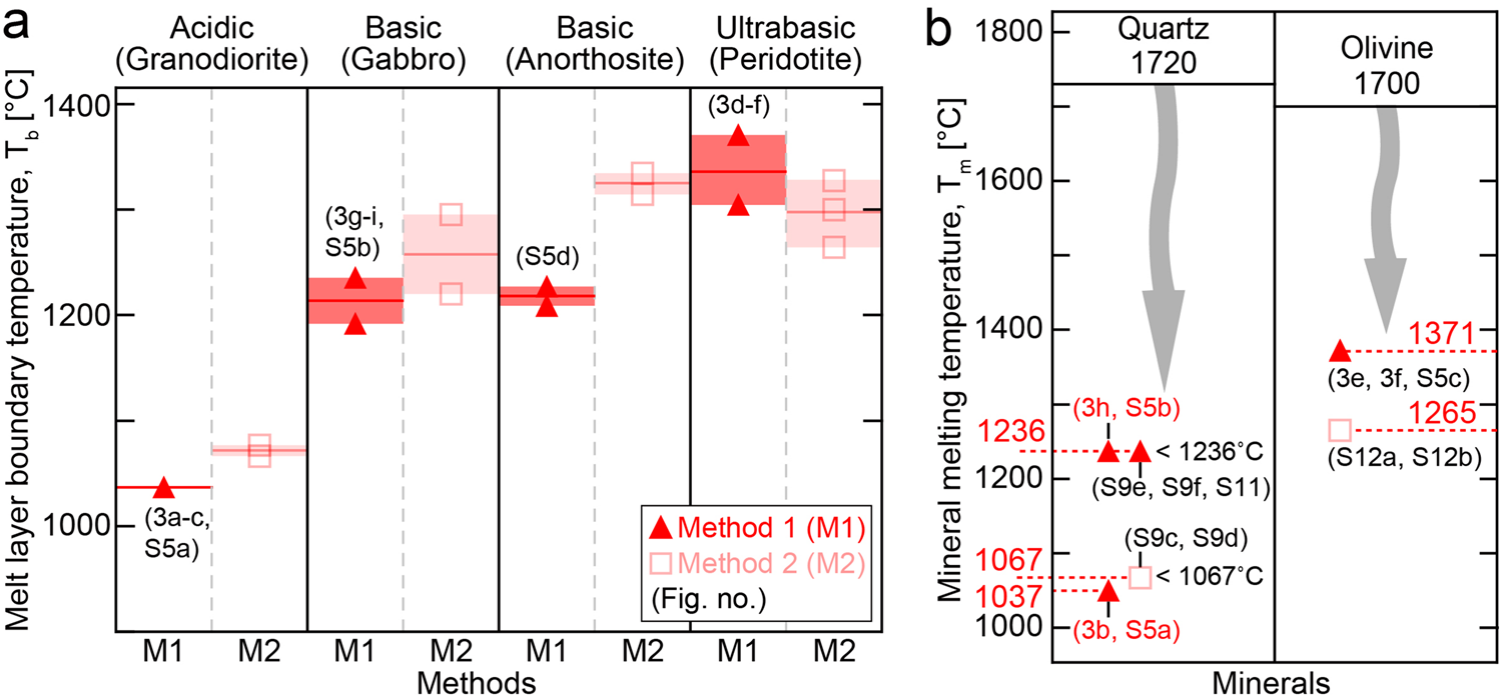
Melting temperatures. (**a**) T_b_ estimated for four types of rocks. (**b**) Comparison of melting temperatures (T_m_) for quartz and olivine determined using slow static heating with that determined by rapid frictional heating (this study). Note the significant T_m_ differences for the minerals. The inequality symbol " < " used with some temperature values indicates that evidence of melting is observed within the wall rock, implying that the melting temperature of minerals is expected to be lower than that at the melt layer boundary. Next to the symbols, the corresponding figure numbers are given.

**Figure 3:**
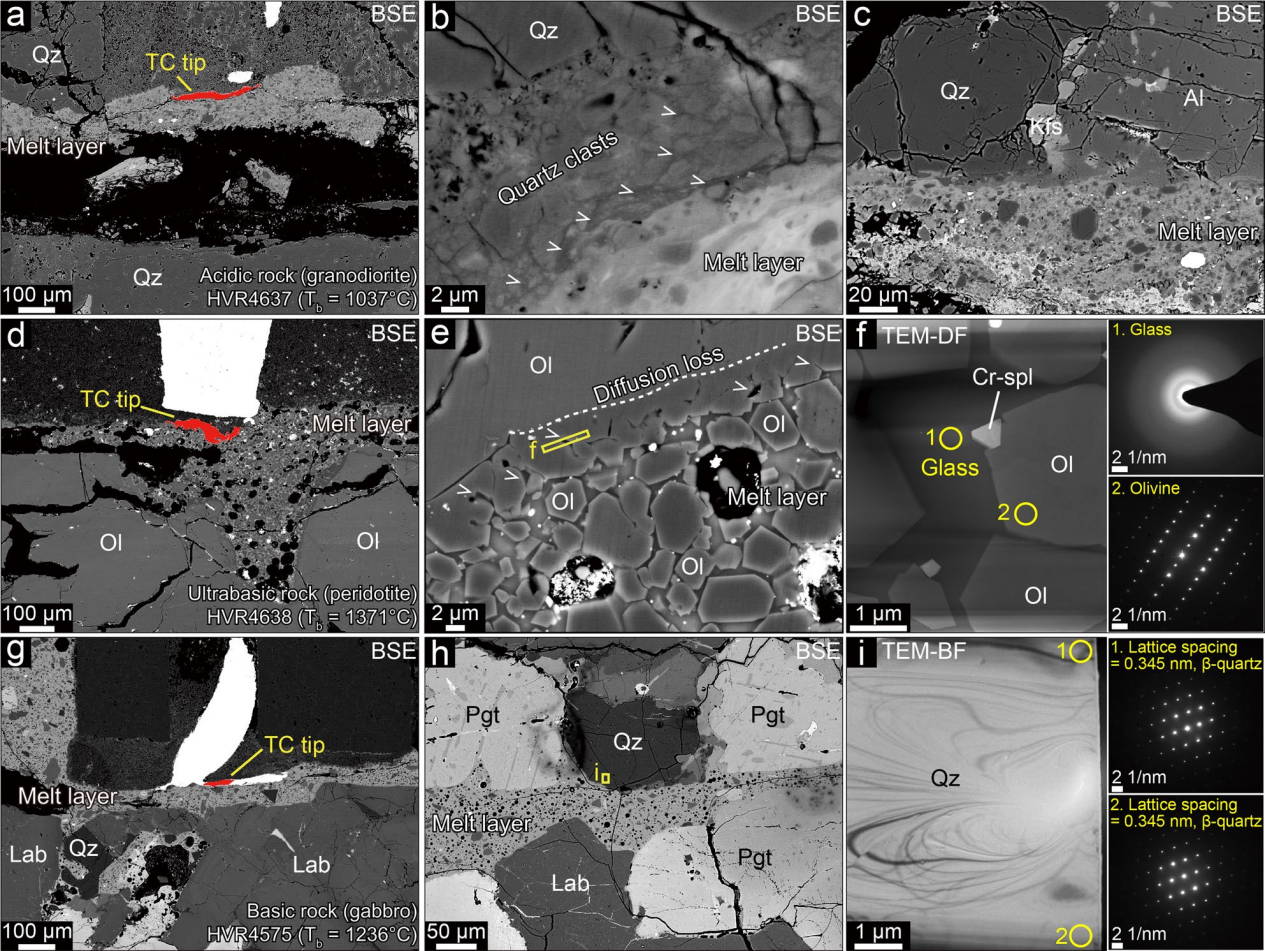
Melting of high-T_m_ minerals. (**a**) Melting in granodiorite (T_b_ = 1037 °C). Note the narrow TC tip (red area) on the melt layer boundary. (**b**) Host rock–melt boundary area, where silica glass (indicated by ‘v’) between quartz clasts in the host rock indicates quartz melting at < 1037 °C (see also Figs. S5a, b and S6a–c). (**c**) Coexisting quartz and albite along the nearly flat melt boundary, indicating their melting at 1037 °C. (**d**) Melting in peridotite (T_b_ = 1371 °C). TC tip indicated by red area. (**e**) Newly crystallized olivine with straight boundaries—not only in the melt layer, but also along the host rock boundary. Note the glass (‘v’) along the host rock boundary. (**f**) Glass (1) and olivine (2) in the boxed area in (**e**) confirmed by TEM observation. Thus, T_m_ (olivine) = T_b_ = 1371 °C. (**g**) Melting in gabbro (T_b_ = 1236 °C). TC tip indicated by red area. (**h**) Coexisting quartz and pigeonite along the nearly flat melt boundary. (**i**) β-quartz in the boxed area in (**h**) confirmed by TEM observation. Thus, T_m_ (β-quartz and pigeonite) = T_b_ = 1236 °C. Qz, quartz; Al, albite; Ol, olivine; Cr-spl, chrome-spinel; Kfs, K-feldspar; Pgt, pigeonite; Lab, labradorite. BSE, back-scattered electron image; TEM-DF, transmission electron microscope dark field image; TEM-BF, transmission electron microscope bright field image.

During our experiments, temperature was measured with K- and R-type thermocouples (TC) with different measurement ranges (Fig. 1; see “Methods”). The melt layer boundary temperature (T_b_) was then estimated using two different methods, based on the measured temperatures and microstructural observations (see details in “Methods”). In Method 1, T_b_ was directly measured by a TC, which was initially set nearly at the same level as the fault surface (Fig. 1c; Fig. S4), while Method 2 involved the estimation of T_b_ using both the TC temperatures, measured at different distances from the melt layer, and a shortening rate measured in the frictional melting experiment (Fig. 1d; ref.^25^). T_b_ data are shown in Fig. 2a; Table S3. Out of the methods, Method 1 was established as yielding more reliable T_b_, as we were able to confirm, using an electron microscope, that the TC was located right at the boundary of the melt layer (Fig. 3; Fig. S4). In Method 2, there are some uncertainties of thermal diffusivity and TC locations, so the T_b_ values from the method are less reliable than those from Method 1. The T_b_ estimated by both the methods appeared to increase as the silica content of the rocks decreased: Method 1 data showed an increase in T_b_, from 1037 °C (granodiorite) to 1305–1371 °C (peridotite) (Fig. 2a). The T_b_ values established for each rock using Method 1 were lower than the T_m_ previously reported for quartz (~ 1730 °C) and olivine (~ 1700 °C) (Fig. 2; ref.^9,26^).

### Microstructural observations and estimation of T_m_

It was then a question whether the established T_b_ values supported the selective melting of low-T_m_ minerals (e.g., only the biotite and amphibole in granodiorite). To explore this question, we performed detailed microstructural and chemical analyses, using electron microscopy (see “Methods”). In granodiorite and gabbro, we observed quartz clast–glass aggregates, which probably evolved from severely fractured quartz grains, very close to the melt layer boundary (Fig. 3a, b, g–i). Transmission electron microscope (TEM) observation confirmed that the quartz clasts included both α- and β-quartz, down to a few tens of nm in size, and there was silica glass between them (Figs. S5a, b and S6). This result showed that fractured quartz could be melted at a much lower temperature (< 1037 °C; Fig. 2) than its conventionally used T_m_ (~ 1720 °C).

In the peridotite melt layer, olivine clasts, euhedral olivine grains (< 5 µm in length), and glass were observed (Fig. 3d–f; Fig. S5c). The euhedral olivine grains may have originated from the melting of either olivine or pyroxene in the peridotite (Table S5). The characteristics of newly crystallized, euhedral olivine grains, created during frictional melting, have been known from previous studies on natural and experimental pseudotachylytes derived from the same peridotite^10,27^ (1) it has less FeO, and more Cr_2_O_3_, Al_2_O_3_ and CaO, than the olivine clast; and (2), it is generally non-zoned. Based on the characteristics, we were able to identify newly grown, euhedral olivine grains in the experimental melt layer (Fig. S7 and Table S4). The original melt chemistry in our specimens was then inferred from the newly crystallized olivine and the residual glass. Our chemical analysis, using an electron microprobe on the glass and the euhedral olivine grains, showed that the chemistry could be explained if 53 wt% of the materials came from olivine (Tables S5 and S6) —which strongly supported the melting of olivine during the experiments. The idea that olivine had melted at the host rock–melt layer boundary was further supported by the occurrence of a narrow zone—which was characterized by olivine grains with straight boundaries, interstitial glass between the olivine grains, and diffusional element loss—along the boundary (Fig. 3e, f; Fig. S5c). This result showed that olivine had melted at temperatures (< 1305–1371 °C) much lower than its previously known T_m_ (~ 1700 °C) (Figs. 2 and 3d; Table S3).

In addition to the quartz and olivine, which had been known as high-T_m_ minerals, labradorite in the gabbro and anorthosite, albite in the granodiorite, K-feldspar in the granodiorite and gabbro, enstatite in the gabbro and peridotite, pigeonite in the gabbro, and diopside in the gabbro and peridotite appear to have melted at temperatures much lower than we expected; there is uncertainty in discussing T_m_ based on T_b_ values estimated by Method 2, but an interesting finding is that the minerals are melted in the wall rocks near the melt layer boundary at temperatures lower than T_b_ if those are severely comminuted, or those react together (Figs. S9–S12).

## Discussion

**Figure 4:**
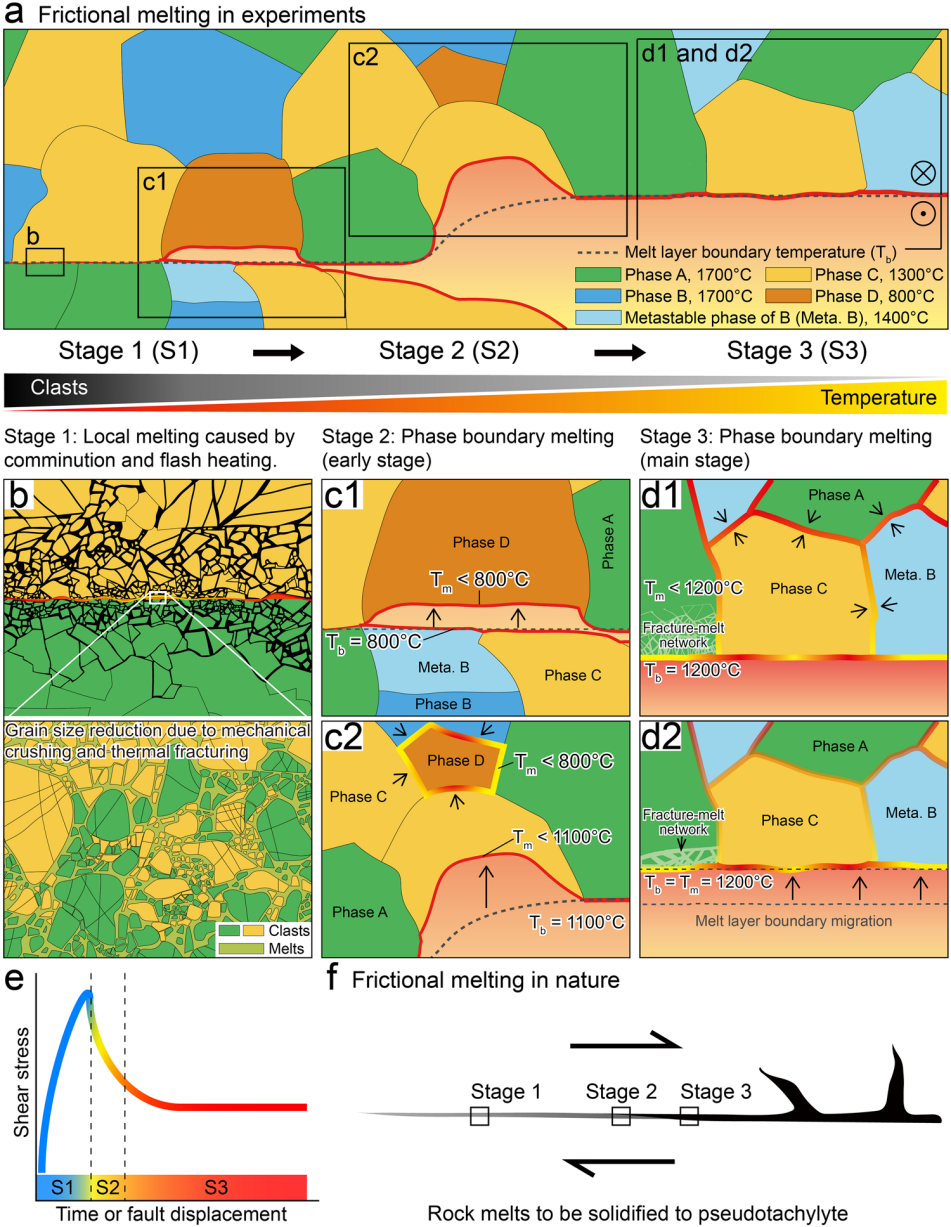
Melting mechanisms during rapid slip. (**a**) Schematic illustration showing frictional melting in an experimental fault. Five phases (Phases A to D, and a metastable phase of B) with different T_m_ are shown. Dextral strike-slip faulting is assumed. (**b**) Local melting caused by grain size reduction, with dominant nanoparticle melting at temperatures much lower than their T_m_ (flash melting may also be possible). (**c**) Phase boundary melting (early stage). Here we see dominant melting of low-T_m_ minerals at temperatures lower than their known T_m_. The melting of Phase D at the fault surfaces (c1) is followed by the melting of Phase C at the host rock–melt layer boundaries (c2), and the melting of Phase D at its boundaries with other solids in the host rock (c2), with increasing temperature. A rough melt-layer boundary exists, due to the dominant melting of relatively low-T_m_ minerals (Phases C and D). (**d**) Phase boundary melting (main stage). Here, we see extensive melting along the solid phase boundaries (black arrows) and fracture network in the host rock (d1), and along the host rock–melt layer boundary (d2), at temperatures (T_b_) lower than their T_m_. At this stage, as the minerals are melted at nearly the same temperature, a relatively smooth melt layer boundary is apparent. Note the melting of Meta. B at temperatures lower than its metastable T_m_. (**e**) Stages of fault slip correlated on a shear stress-displacement curve. (**f**) Change in dominant melting mechanisms with increasing displacement, on a natural fault showing the dextral sense of strike-slip.

To understand why the rock-forming minerals (quartz and olivine) melted at much lower temperatures than previously thought, the dominant melting mechanisms that apply during seismic slip were considered. During the early stage of slip, mechanical rock crushing and thermal fracturing occur effectively on fault surfaces (Stage 1), as shown in Fig. 4a, b^14,16,28–30^. These processes may induce significant grain size reduction and the formation of nanoparticles, which are known to melt at much lower temperatures than their bulk T_m_^15,31^. The quartz clast–glass aggregates in Fig. 1b–d of^15^ may be an example of this type of mechanism in operation. Based on the inferred melting temperature of severely fractured grains in our specimens, such as those shown in Fig. 3a, b; Fig. S5a, b, we expect quartz to melt at temperatures even < 1000 °C due to this mechanism. Flash melting due to localized heating at sliding contacts^17,32–34^ may also occur during Stage 1, regardless of how much T_m_ reduction occurs.

With further displacement, slip zone temperatures rise high enough for low-T_m_ minerals to be melted (Stage 2) as shown in Fig. 4a, c. In a process which differs from what has been assumed previously, the minerals melt not at their known T_m_ but at lower temperatures, in a phenomenon which seems to be due to their chemical interactions with other neighboring phases. Due to the dominant melting of low-T_m_ minerals, the host rock–melt boundary may be rough (Fig. S8). With further displacement, the temperature in and around the slip zone rises more, and then extensive melting becomes possible (Stage 3), as shown in Fig. 4a, d. Melting occurs at temperatures lower than their T_m_ along the solid phase boundaries and fractures in the host rock near the melt layer (Fig. 4d1; Figs. S9 and S10), and almost all minerals—including metastable phases (e.g., β-quartz)—melt at similar temperatures along the melt boundary (Figs. 3c, h and 4d2; Fig. S9a, b). Based on these outcomes, we form the view that phase boundary reactions are fast enough for minerals to be melted at much lower temperatures than their known T_m_, in as little as a few seconds. This idea is incompatible with the selective, disequilibrium melting of low-T_m_ minerals, but is more consistent with the quasi-equilibrium melting model, in which melting at mineral interfaces can be considered as an example of the contribution from equilibrium thermodynamics to lowering T_m_^15^.

It can also be seen that large β-quartz grains with few fractures, melt along the host rock–melt layer boundary, at 1236 °C (Fig. 3g–i), and along the boundary with labradorite, at < 1236 °C (Figs. S9e, f and S11), unlike the metastable melting of β-quartz alone, at ~ 1400 °C^15,35,36^. This demonstrates that there is little contribution from metastable melting, if at all, in multi-phased rocks. In our model, whether minerals are metastable is not critical, with phase boundary reactions the common mechanism for the observed T_m_ reduction. In Stage 3, due to the melting of multiple phases, the melt chemistry becomes more complicated, and the melt layer boundary becomes smoother than before (Fig. 4d). At this stage, T_b_ (and also T_m_ of the phases at the host rock–melt layer boundary) may not be constant, but rather change, existing through a range that depends on the chemical evolution that takes place along the host rock–melt boundary. This indicates that, as rapid sliding proceeds in a natural fault, the dominant melting mechanism at any specific location may change with time (Fig. 4e, f). As reported from the observations of natural and experimental pseudotachylytes ^37–40^, the water either initially present in fault slip zones or generated due to mineral decomposition during slip may be involved in melting reactions, possibly reducing the melting temperatures of grains^41,42^. In our experiments, water in room air and newly released water from dehydrated minerals probably worked in Stage 1, and Stages 2 and 3, respectively. The decrease in melting temperature in Stages 2 and 3 is thought to be partially due to the involvement of water in the melting reaction^15^.

Our finding from the experimental study may be useful for interpreting the mechanics of seismic slip of natural faults using the microstructures of pseudotachylytes. For instance, the new estimates of T_m_ may constrain a more reliable temperature range that can be used in the estimations from natural pseudotachylyte-bearing faults of melt viscosity^38,43,44^, coseismic fault strength and fault displacement^45,46^, heat penetration depth near a melt layer^47^, and frictional heat and earthquake energy budget^48^. It may also apply to the mechanical models of large landslides and caldera collapses, since the evidence of frictional melting has been observed from the dynamic events^2–6,49^.

## Methods

### Starting materials

Four igneous rock types—granodiorite (Gyeongju, Korea), gabbro (Belfast, South Africa), anorthosite (Sancheong, Korea), and peridotite (Balmuccia, Italy) —were used in the study. Solid cylinders were prepared from the rocks by coring. After polishing, the end surfaces of the rock cylinders were observed, using an electron microscope. By applying Adobe Photoshop to back-scattered electron images taken from the cylinders, we created false-color images showing mineral distribution—and then analyzed these, to identify the mineral modes present in the rocks. The areas occupied by rock-forming minerals were measured, using NIH Image J software, and then the modes of the minerals were calculated (Fig. S1 and Table S1).

### Specimen configuration

Solid rock cylinders 25 mm in diameter and ~ 20 mm high were prepared, using a cylindrical grinder, and their end surfaces were ground with #100 SiC powder. For each run, a pair of cylinders (one rotating, the other stationary) were used (Fig. 1a). The cylinders were wound by a stainless steel wire, to prevent significant rock failure caused by thermal fracturing during the experiments (Fig. 1b). Holes for thermocouples (TC) were made in the stationary rock cylinders, using a micro-drilling machine equipped with ultrahigh-strength, TiAlN-coated (titanium-aluminum nitride) micro-drill bits. The holes were located 5 and 6 mm (for K-type and R-type TCs, respectively) from the periphery of the stationary rock cylinder, and were 1.3 mm and 3.3 mm in diameter, respectively. The tip of the K-type TC was formed by welding the positive and negative TC wires, using a portable arc welder with a carbon electrode, while that of the R-type TC was created using argon welding. A TC was installed in each hole, and filled with a ceramic bonding preparation, and then cured for 1 day.

### Frictional melting experiments (high-velocity rotary shear tests)

All shear tests were conducted using a high-velocity, rotary shear apparatus (HVR), as previously described^50^, and applying the experimental conditions listed in Table S2. Mechanical and temperature data were collected at sampling rates of up to 500 Hz, while in some runs on peridotite, an R-type TC GL840 (GRAPHTEC) data logger was used, with a sampling rate of 5 Hz. To obtain both temperature data and sheared experimental specimens successfully, the duration of the tests was determined using trials. Shear tests involving K-type TCs were stopped before the temperature reached the maximum K-type TC temperature (~ 1259 °C), whereas tests employing R-type TC could be run for larger displacements, due to the tip size of R-type TC being larger than that of the K-type TC and its higher maximum measurable temperature (~ 2500 °C). For the K-type TC (wire diameter of 0.127 mm, OMEGA), a beaded-type tip was made by twisting and then welding using a portable arc welder. As for the R-type TC (wire diameter of 0.511 mm, custom-made), a beaded-type tip was prepared by argon welding.

### Microstructural observation and chemical analysis

Microstructural and chemical analyses on the starting materials and recovered sheared specimens were conducted at the Center for Research Facilities, Gyeongsang National University, Korea, using a field-emission electron probe micro-analyzer (FE-EPMA; JEOL-8530F plus), equipped with both energy dispersive and wavelength dispersive X-ray spectrometers (EDS and WDS). All BSE images were captured using the FE-EPMA, while quantitative chemical analyses were conducted with a 12-element WDS, with a 15-kV acceleration voltage, and a 10-nA current.

Specimen preparation for transmission electron microscopy (TEM) was performed with two focused ion beam (FIB) instruments—a Quanta 3D FEG, at the Korea Basic Science Institute (KBSI), and a Helios NanoLab 600, at the Korea Institute of Science and Technology (KIST), Korea. TEM observation and chemical analyses were conducted on 5 × 7 µm^2^, < 100-nm thick specimens, using an electron microscope (F20 G2, Tecnai) equipped with an energy dispersive X-ray spectrometer (EDAX; PV9761 Si(Li) detector), at an acceleration voltage of 200 kV (also at KIST).

### Estimation of melt layer boundary temperatures (T_b_) and mineral melting temperatures (T_m_)

For melt layer boundary temperature (T_b_) estimation during frictional melting, we used two methods, using a K-type TC and R-type TC (Fig. 1c, d). The maximum temperatures that can be measured by the K-type TC (OMEGA, TT-K-35-SLE) and R-type TC (OMEGA, SP13R-020) are ~ 1259 °C and ~ 2500 °C, respectively. The limits of error are 2.2 °C or 0.75% (whichever is greater) for the K-type TC, and 1.5 °C or 0.25% (whichever is greater) for the R-type TC. TC can be used to measure temperatures at selected areas inside rock cylinders^51–53^. The details of the methods used are given below.

#### Method 1

This is a direct T_b_ measurement method, which involves using a TC with its tip located at the same level as the experimental fault surface (Fig. 1c). The method aims to measure the temperature at an area either along the melt layer boundary or within the melt layer without TC failure by the end of the experiment. The smaller the TC tip, the better the output, as a local temperature.

Previously, direct temperature measurements were tried; the temperature recorded at the time of TC failure (or the value just before abnormal fluctuations of TC output) was thought to be either a slip zone or a melt layer boundary temperature^8^. In these cases, though, the size and exact location of the temperature-measured area were unknown, as these were not examined by microstructural observation. In our study, we found that TC tips installed at the same level as the fault surface could survive in most (not all) experiments. We then made the TC tips as small as possible, by grinding the fault surfaces with #100 SiC powder (Fig. S3). To prevent not only TC failure but also the temperature rising to the maximum measurable by the TC, the experiment (on the granodiorite, in particular) had to be short, stopping after a few seconds (Fig. S2). At a high normal stress—for example, at 10 MPa—this short duration was sufficient for a continuous melt layer to be formed along the slip zone. After the experiments, the recovered specimens were cut, polished, and then observed under an electron microscope for the determination of the location of the TC tip within the specimens.

The positive and negative wires of a K-type TC consist of chromel (Ni and Cr) and alumel (Ni, Mn, Al, and Si), respectively, while those of an R-type TC consist of Pt–Rh and Pt, respectively. By mapping the constituent elements, we could clearly outline the real tip area, where the positive and negative wires were welded (Fig. S4). If measuring is performed following this procedure, Method 1 can be the most reliable way to measure the T_b_.

#### Method 2

This method, used as an auxiliary approach to estimating T_b_ in the study, is based on an analytical solution of the temperature profile in a solid, near a melt layer (Eq. 1; for details, see Section "Frictional melting experiments and temperature measurements" in^25^).

1 $$T=\left({T}_{b}-{T}_{i}\right){e}^{-\xi \frac{v}{\kappa }}+{T}_{i}$$

In Eq. (1), T_b_ represents the temperature at the melt layer boundary, with T_i_ standing for the initial temperature in the rock (room temperature, in our study). ξ represents the distance from the melt layer boundary to a place in the solid (the location of TC in our study), ν stands for a shortening rate, which is usually constant at a steady-state (i.e., melt production rate = melt removal rate), and κ represents the thermal diffusivity of the solid. If we measure the temperatures (two T values) using two TC at the different distances (two ξ values) during a frictional melting experiment, and calculate the shortening rate using the plot of axial displacement against time, we can calculate two unknown values (T_b_ and κ) by solving a system of two equations. For ξ, the distance was measured from the center of each TC to the melt layer boundary in the BSE image, whereas for a rough melt layer boundary, an average distance was taken. Where there are only small ξ differences, the local melt layer boundary topography may affect the estimated T_b_ significantly—indicating that for reliable T_b_ estimation, it is better to have a large distance between two TCs. However, for a large difference in the distance, thermal diffusivity cannot be assumed to be the same in the areas around the two TCs. Given the uncertainty in ξ and κ values, Method 2 would yield less reliable values of T_b_ than those by Method 1.

Based on the estimated T_b_ (Fig. 2a), and on detailed microstructural observations, rock-forming mineral melting temperatures (T_m_) were estimated (Fig. 2b). In estimating T_m_, T_b_ values established using Method 1 were used preferentially, as they were more reliable than those by Method 2. The T_m_ references for quartz and olivine shown in Fig. 2b were ref.^9^ and ref.^26^, respectively.

#### Effect of the sampling rate and response time of thermocouples on the measured temperature

We examined the experimental data to see how the sampling rates of the recorders used in the experiments and response times of the K-type and R-type TCs affected the measured temperature. The response time is defined as the time required to reach 63.2% of an instantaneous temperature change^54^. The response times calculated for air at room temperature and atmospheric pressure moving with a velocity of 20 m/s for thermocouples are available^54^. However, it is uncertain whether the response times can be applied to the conditions involving a rapid temperature increase of over 1,000 °C within a few seconds during the high-velocity friction experiments at high normal stresses. Thus, we estimated the TC’s response times based on our experimental data.

In an experiment on gabbro (HVR4575) where a K-type TC was used, a data sampling rate of 500 Hz was sufficient enough to record details of temperature change, considering the data showing that there was no significant temperature variation in the last stage of the experiment (Fig. S13a, b). 0.02 s after the onset of deceleration at the end of the experiment, the measured temperature began to decrease from the maximum temperature. Thus, the upper bound of K-type TC response time (t_R_) was 0.02 s, which was short enough not to cause a significant underestimation of T_b_ (e.g., 1.8 °C; Fig. S13b). In an experiment on peridotite (HVR4638) where an R-type TC was used, a low sampling rate of 5 Hz was used because the performance of the data logger for the R-type TC allowed only that level of the sampling rate. Despite this, the low sampling rate for temperature data appears to have caused only a slight underestimation (1.3 °C) of the maximum temperature, which was reached at the end of the slip (or at the onset of slip deceleration) (Fig. S13c, d). The upper limit of t_R_ of the R-type TC is the time elapsed from the moment the deceleration of slip began, that is, from the point at which the maximum temperature was reached, to the point at which the decreased temperature was first recorded. Thus, the t_R_ is 0.04 s (Fig. S13d). After reviewing all the temperature data, it was found that the t_R_ for the K-type and R-type TCs are 0.02‒0.06 s and 0.04 s, respectively. In our experiments, possible underestimation of T_b_ due to the sampling rate or TC response time was 2.0‒20.4 °C when measured by the K-type TC and 1.3 °C when measured by the R-type TC.

#### Effect of the size reduction of TC tips on the measured temperature

The temperature measurement with thermocouples is based on the Seebeck effect^55^. The effect describes a thermoelectric phenomenon in which temperature differences between two dissimilar metals or semiconductors in a circuit are related to an electrical voltage. The Seebeck coefficient, given in volts per kelvin (V/K), of a material is a measure of the magnitude of an induced thermoelectric voltage in response to a temperature difference across that material. For accuracy, using materials with a Seebeck coefficient that is stable over time is desirable^56^. A standard industrial product of thermocouples with a wire diameter has a calibrated Seebeck coefficient. In our experiments, the thermocouples’ tip was observed to be ground and plastically deformed, reducing its size significantly to thinner than 100 μm (Fig. 3a, d, g; Fig. S4). It was uncertain whether the deformed thermocouple tip could show the same Seebeck effect as the original thickness of the wires. Thus, we conducted a temperature measurement experiment with different types of thermocouple tips (a beaded type of 400 μm in diameter, a flattened type of 100 μm in thickness, and a flattened type of 250 μm in thickness). The flattened types of thermocouple tips were made by pressing the beaded tip. We put the thermocouples into a furnace (Nabertherm GmbH, L3/11). We heated it to 500 °C with a heating rate of 500 °C/h, kept the temperature for 30 min for the temperature in the furnace to be uniform, and then checked the temperature outputs from the thermocouples. Then, we heated it to 1000 °C and followed the same procedure. The different thermocouple types in the experiment showed only small temperature differences of 2 to 6 °C (Table S7 and Fig. S14). This observation indicates the deformation and reduction in the size of thermocouple tips during the shear experiments may not affect the measured temperature significantly once they survive the shearing.

## Supplementary Information

Supplementary Information.

## Data Availability

All data are available in the main text or the supplementary materials.
