# Supplementary material for: Frictional melting mechanisms of rocks during earthquake fault slip
Source: Sci Rep. 2023 Aug 2;13:12563. doi: 10.1038/s41598-023-39752-9 (PMC10397195; doi:10.1038/s41598-023-39752-9)
Supplement: Supplementary file 1 — Supplementary Information. [file 41598_2023_39752_MOESM1_ESM.pdf]

Supplementary Information for

Frictional melting mechanisms of rocks during seismic slip

Sangwoo Woo, Raehee Han*, Kiyokazu Oohashi

*Correspondence to: raeheehan@gnu.ac.kr

This PDF file includes:

Fig. S1. The four igneous rocks used in the study.
Fig. S2. Representative frictional behaviors during rotary shear tests.
Fig. S3 Image showing TC installed in an experimental specimen for Method 1.
Fig. S4 Close-up images of TC tips.
Fig. S5 Microstructural evidence for the melting of quartz, olivine, and labradorite.
Fig. S6 TEM observation of severely fractured quartz grains in the host rock near the melt layer boundary.
Fig. S7 Chemical composition across olivine grains.
Fig. S8 BSE images showing the dominant melting of low-T_m mineral.
Fig. S9 BSE images showing melting along fractures and phase boundaries in host rocks.
Fig. S10 BSE images showing melting along the phase boundaries in the wall rock and temperature measured by a thermocouple.
Fig. S11 TEM observation of the boundary area between quartz and labradorite (yellow-boxed area in Fig. S9f).
Fig. S12 BSE images showing melting along the host rock–melt (solid–liquid) boundary.
Fig. S13 Effect of data sampling rate and response time of thermocouples.
Fig. S14 Temperature measurement with different types of thermocouple (TC) tips.
Table S1. Modal mineralogy of the host rocks used in the study.
Table S2. Summary of friction experiments.
Table S3. Melt layer boundary temperatures (T_b).
Table S4. Chemical compositions of olivine in the host rock and melt layer determined by electron microprobe analysis.
Table S5. Chemical compositions of the minerals in peridotite determined by electron microprobe analysis.
Table S6. Chemical composition of glass and newly crystallized olivine, and melting ratios of minerals.
Table S7. Temperature measurement with different types of thermocouple tips.

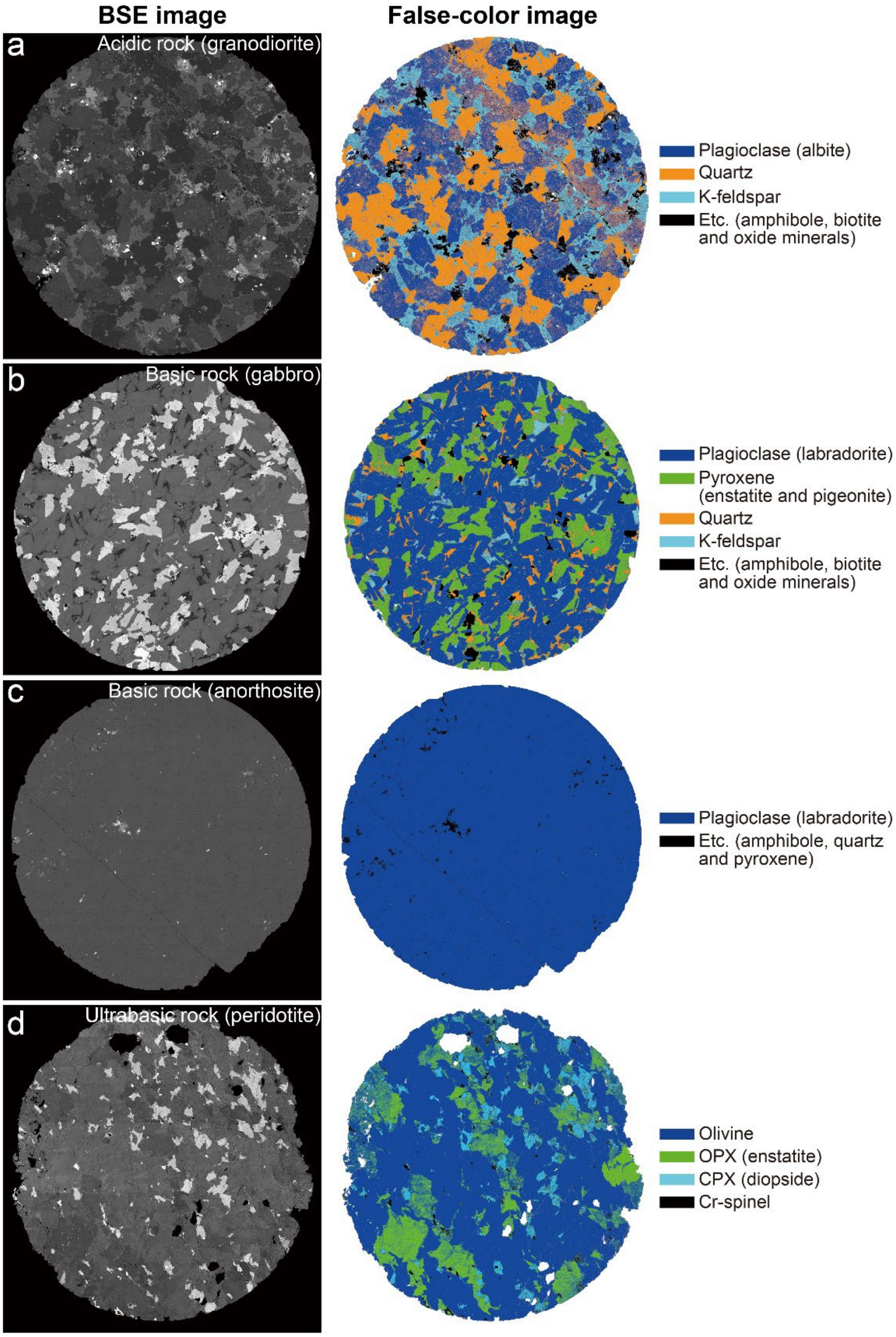

Fig. S1. The four igneous rocks used in the study. a Granodiorite (acidic rock). **b** Gabbro (basic rock). **c** Anorthosite (basic rock). **d** Peridotite (ultrabasic rock). Left side panel: BSE images. Right side panel: false-color images showing the distribution of mineral phases. See also Table S1 for the modes of minerals in the rocks.

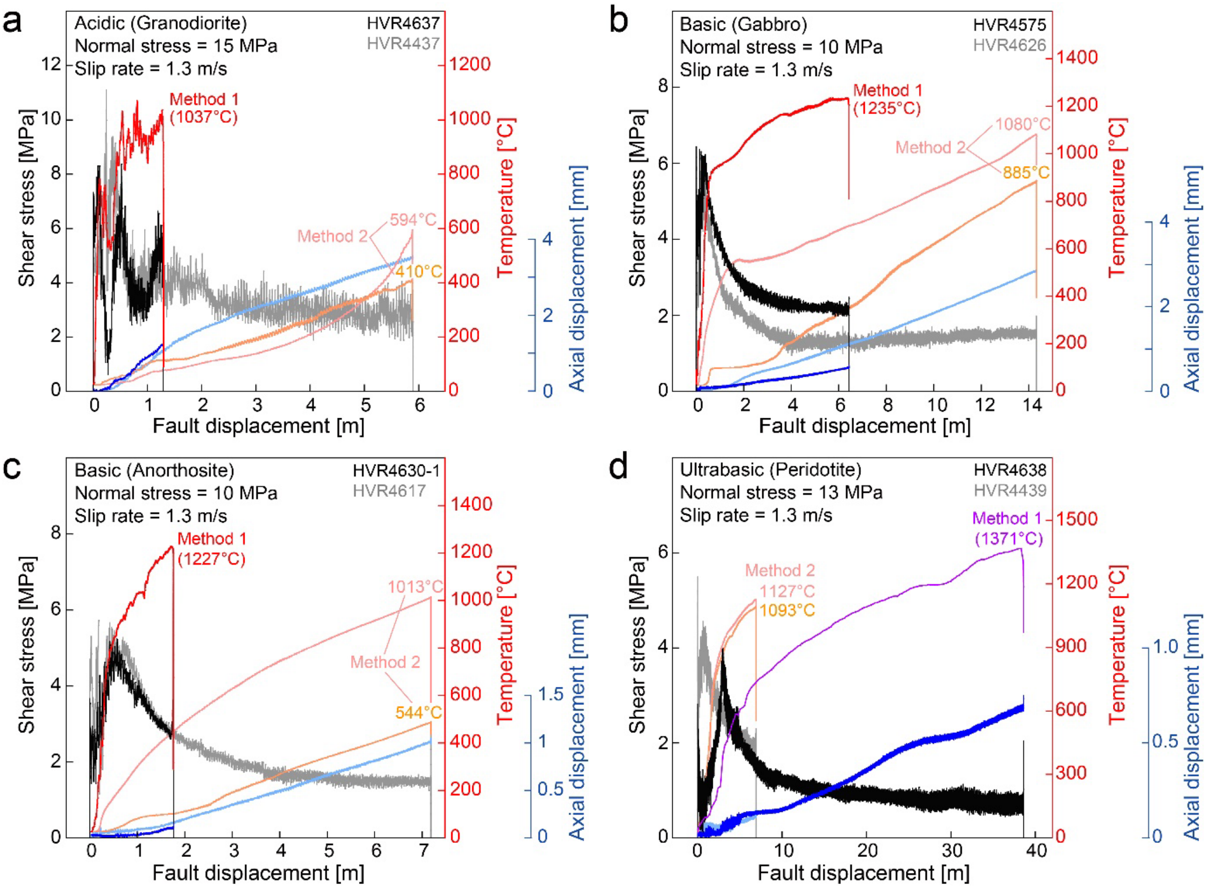

Fig. S2. Representative frictional behaviors during rotary shear tests. a Granodiorite, at a normal stress of 15 MPa. **b** Gabbro, at a normal stress of 10 MPa. **c** Anorthosite, at a normal stress of 10 MPa. **d** Peridotite, at a normal stress of 13 MPa. Black and gray curves: shear stress. Red curve: temperature measured using a K-type TC (Method 1). Purple curve: temperature measured using an R-type TC (Method 1). Pink and orange curves: temperature measured using K-type TC (Method 2). Blue and light blue curves: axial displacement (its increase meaning axial shortening). See also Tables S2 and S3 for the mechanical data and temperatures measured in the experiments. All the tests were conducted at a slip rate of 1.3 m/s.

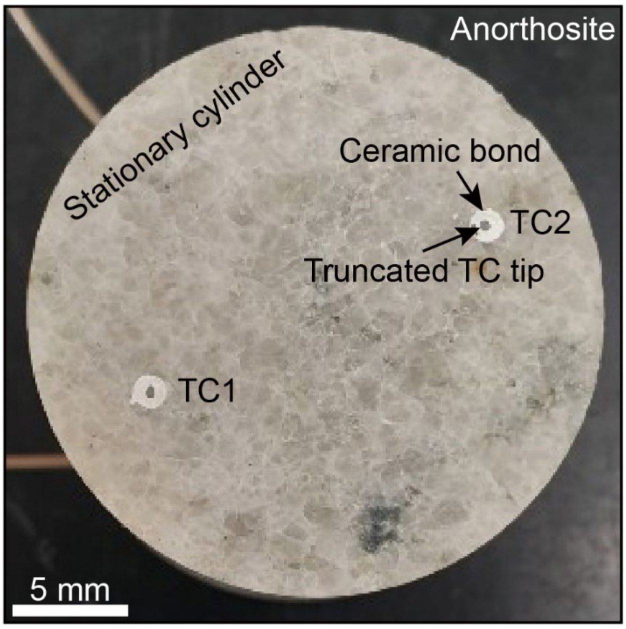

Fig. S3. Image showing TC installed in an experimental specimen for Method 1. The tip of TC was ground down until its upper end was at nearly the same level as the anorthosite experimental fault surface. Note that the truncated TC tip is exposed to the fault surface.

.

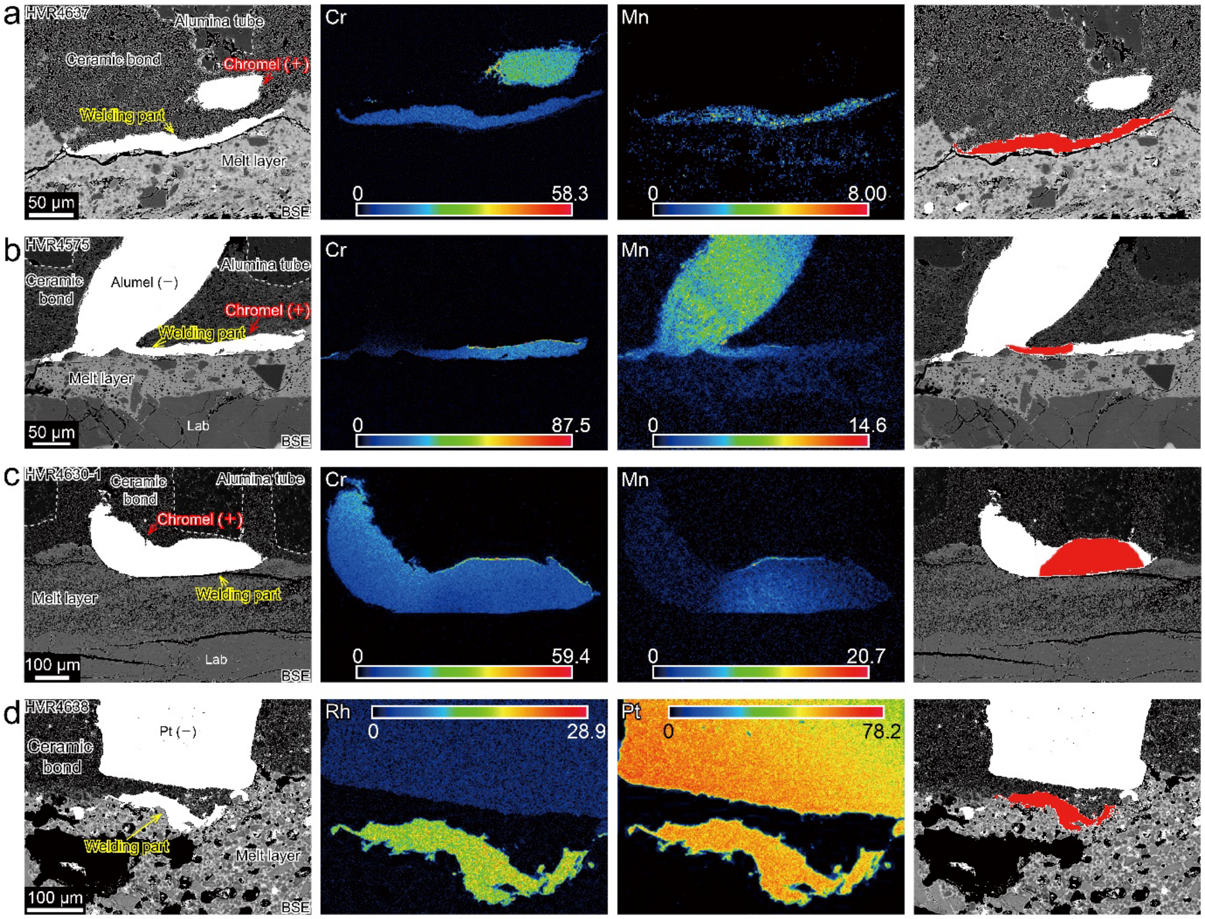

Fig. S4. Close-up images of TC tips. Element mapping can be used to outline the real TC tip area, where the positive and negative TC wires are welded. The K-type TC positive wire is made of Ni and Cr, whereas the negative wire consists of Ni, Mn, Al, and Si. The R-type TC positive wire consists of Pt and Rh, whereas the negative wire is made of Pt. The place where the elements of both positive and negative wires are abundant is defined as the TC tip (red-colored areas in the rightmost column images). Note that the bright area in the BSE image may overestimate the size of a TC tip. Examples shown here represent **a** granodiorite, **b** gabbro, **c** anorthosite, and **d** peridotite. Lab, labradorite.

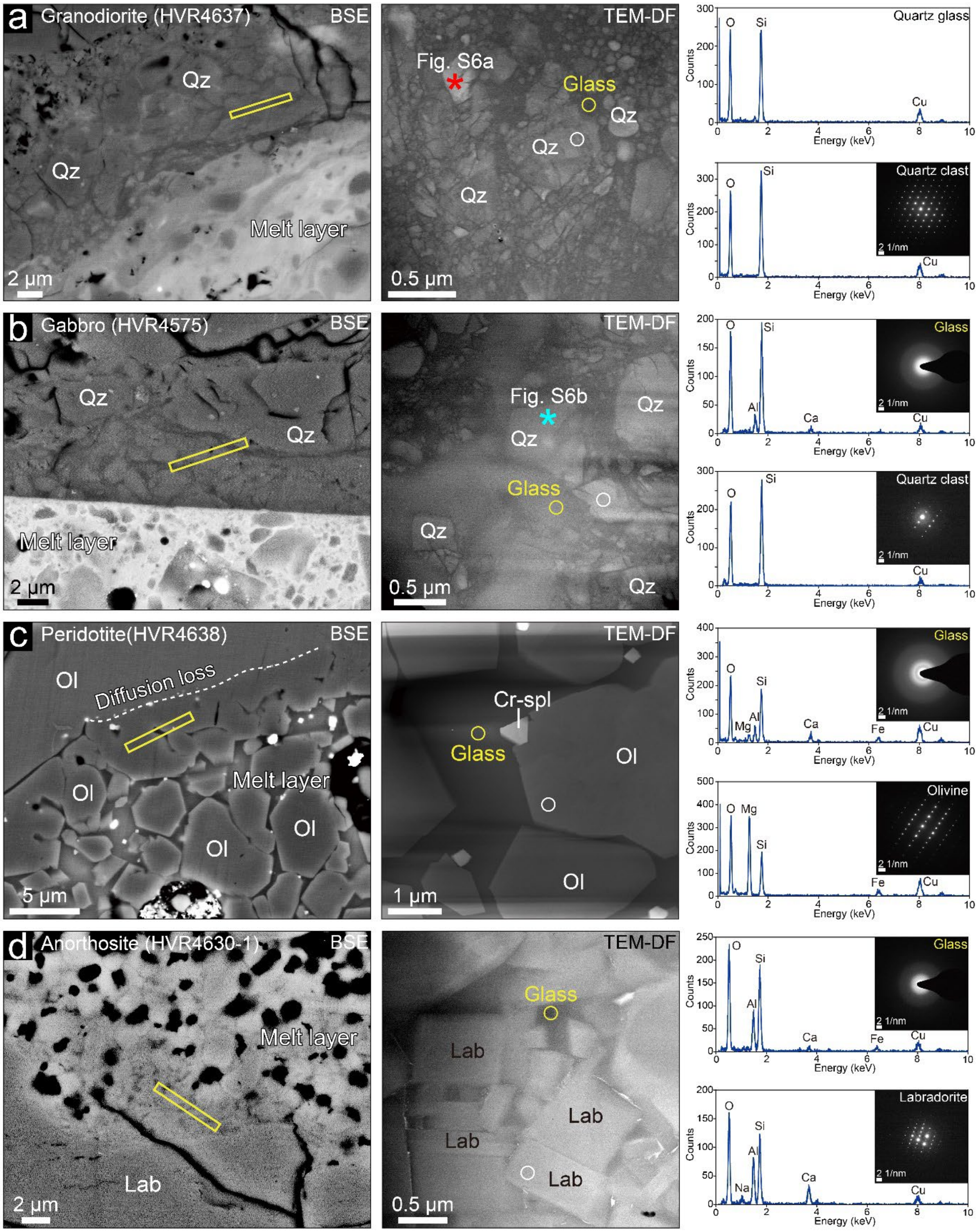

Fig. S5. Microstructural evidence for the melting of quartz, olivine, and labradorite. a Severely fractured quartz grains in granodiorite. From spot analyses in the area, quartz crystals were identified well (right image). Due to beam damage during the analysis, an electron

diffraction pattern could not be obtained in the dark area between quartz clasts (central image). However, a selected area electron diffraction (SAED) pattern obtained from the same area confirmed the coexistence of quartz crystals and glass, indicating that the material between the quartz clasts is glass (Fig. S6)—the red asterisks in this figure and Figs. S6a and S6b indicate the same location. **b** Severely fractured quartz grains in gabbro. It is confirmed by an electron diffraction halo ring pattern (inset in the right image) that the material between the clasts is glass. See also Figs. S6c and S6d. The light blue asterisks in the figures and this figure indicate the same location. Al and Ca peaks in the EDS pattern indicate that materials other than quartz were involved in the melting process in only a minor way (right image). **c** Olivine crystals and glass in peridotite. Note that host rock olivine grains had been melted along the host rock–melt boundary. After this, the new olivine grains, with straight boundaries, crystallized from the melt during cooling, leaving interstitial glass between them (left and central images). The olivine crystals and glass are confirmed using electron diffraction patterns (right image). **d** Labradorite crystals and glass in anorthosite. Along the host rock–melt layer boundary (left image), labradorite grains had been melted, and euhedral crystals of labradorite and glass then formed during cooling (central and right images). In this figure, the left column shows SEM-BSE images, the central column shows TEM-DF images and the right column shows energy-dispersive X-ray spectroscopy (EDS) data and selected area electron diffraction (SAED) images (inset). The yellow boxed areas indicate the sampling location for TEM observation, while the yellow and white open circles show locations where diffraction patterns were taken. Qz, quartz; Ol, olivine; Lab, labradorite; BSE, back-scattered electron image; TEM-DF, transmission electron microscope dark field image.

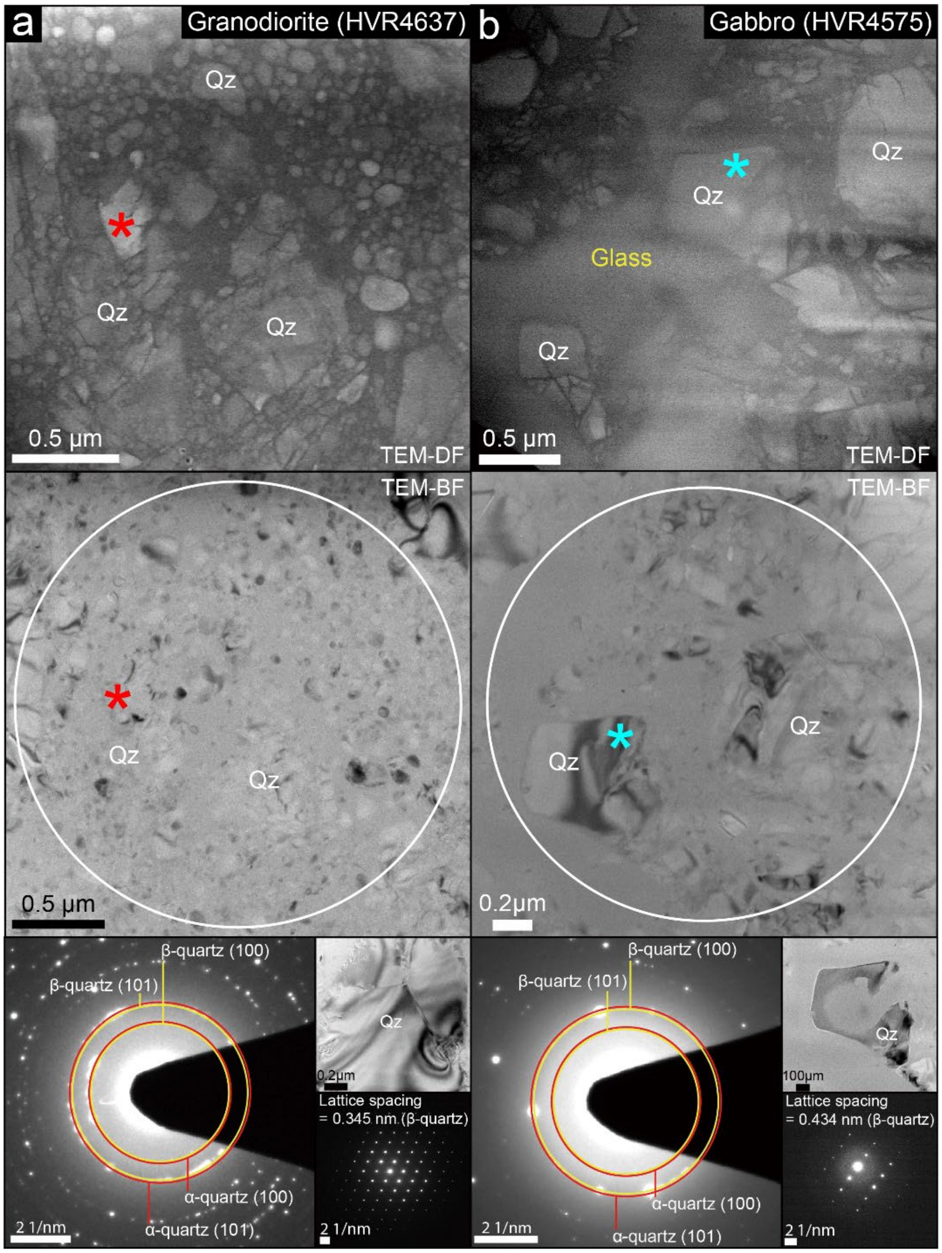

Fig. S6. TEM observation of severely fractured quartz grains in the host rock near the melt layer boundary. Results from **a** granodiorite and **b** gabbro. The red and light blue asterisks in this figure and Fig. S5 indicate the same locations. SAED patterns showing halo features and rings of diffraction spots indicate the coexistence of randomly oriented α- and β-quartz grains and glass (central images). The β-quartz is also confirmed by the lattice spacing in the diffraction patterns (right images). Qz, quartz; TEM-DF, transmission electron microscope dark field image; TEM-BF, transmission electron microscope bright field image.

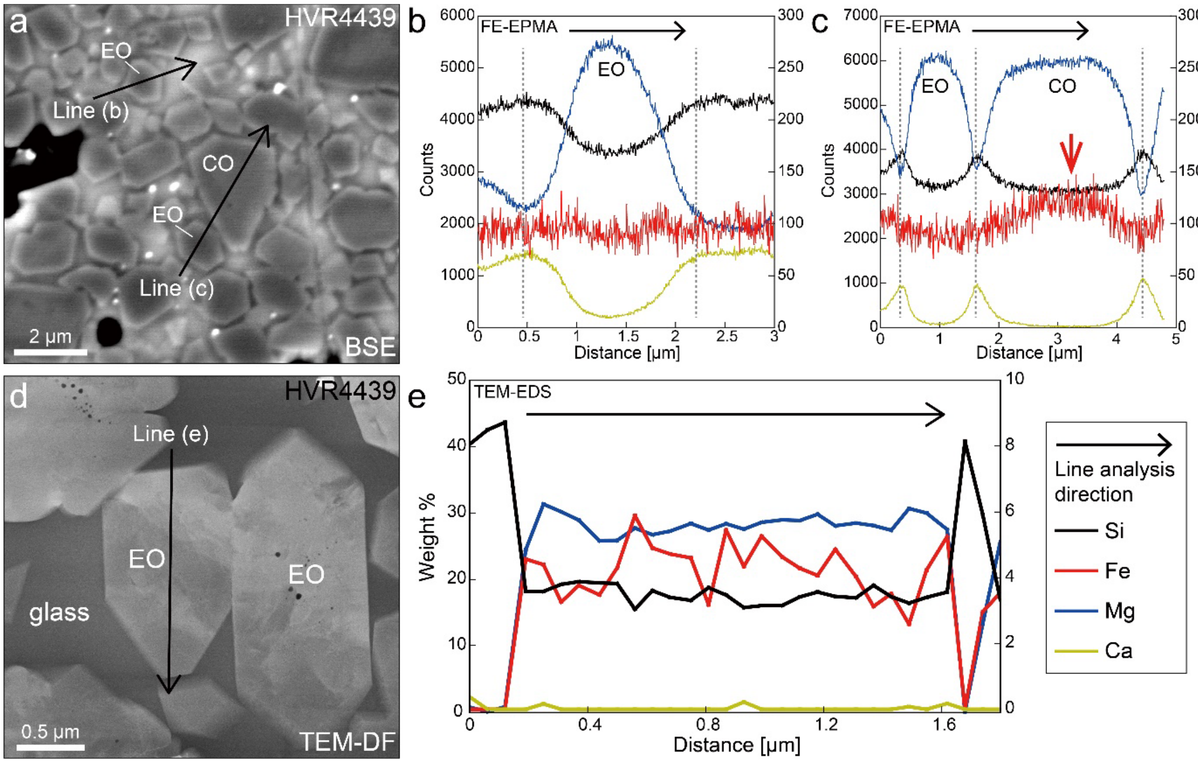

Fig. S7. Chemical composition across olivine grains. a BSE image of the melt layer in peridotite. **b, c** Results of chemical analyses conducted along the two lines shown in **a**. EO, euhedral olivine; CO, olivine clast. Note that the core part of the CO exhibits a higher FeO content (indicated by the red arrow) than the EO crystal does. **d** TEM-DF image showing EO crystals. **e** EDS line analysis across the olivine (indicated by the black arrow in **d**). High FeO content is not observed in the core part. BSE, back-scattered electron image; TEM-DF, transmission electron microscope dark field image.

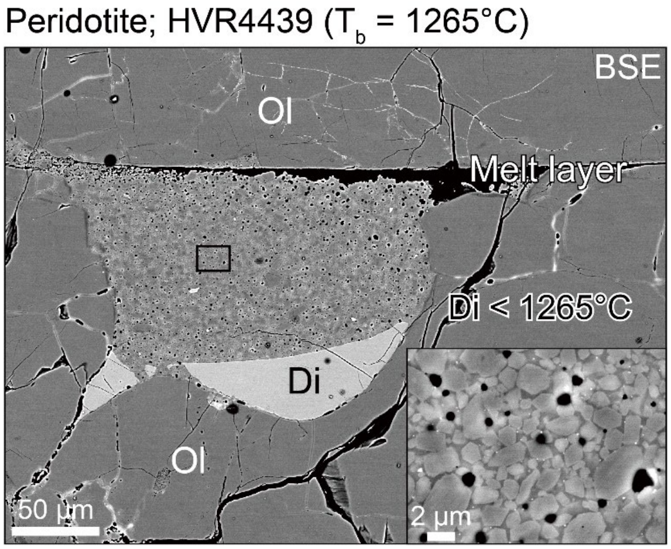

Fig. S8. BSE images showing the dominant melting of low-T_m mineral. Diopside (T_m = 1400 °C) in the peridotite has dominantly melted, and the melt layer boundary is rough. Note that the actual diopside T_m should be < 1265 °C, rather than the widely used T_m of 1400 °C. The inset shows olivine clasts and newly grown olivine crystals. Ol, olivine; Di, diopside. BSE, back-scattered electron image.

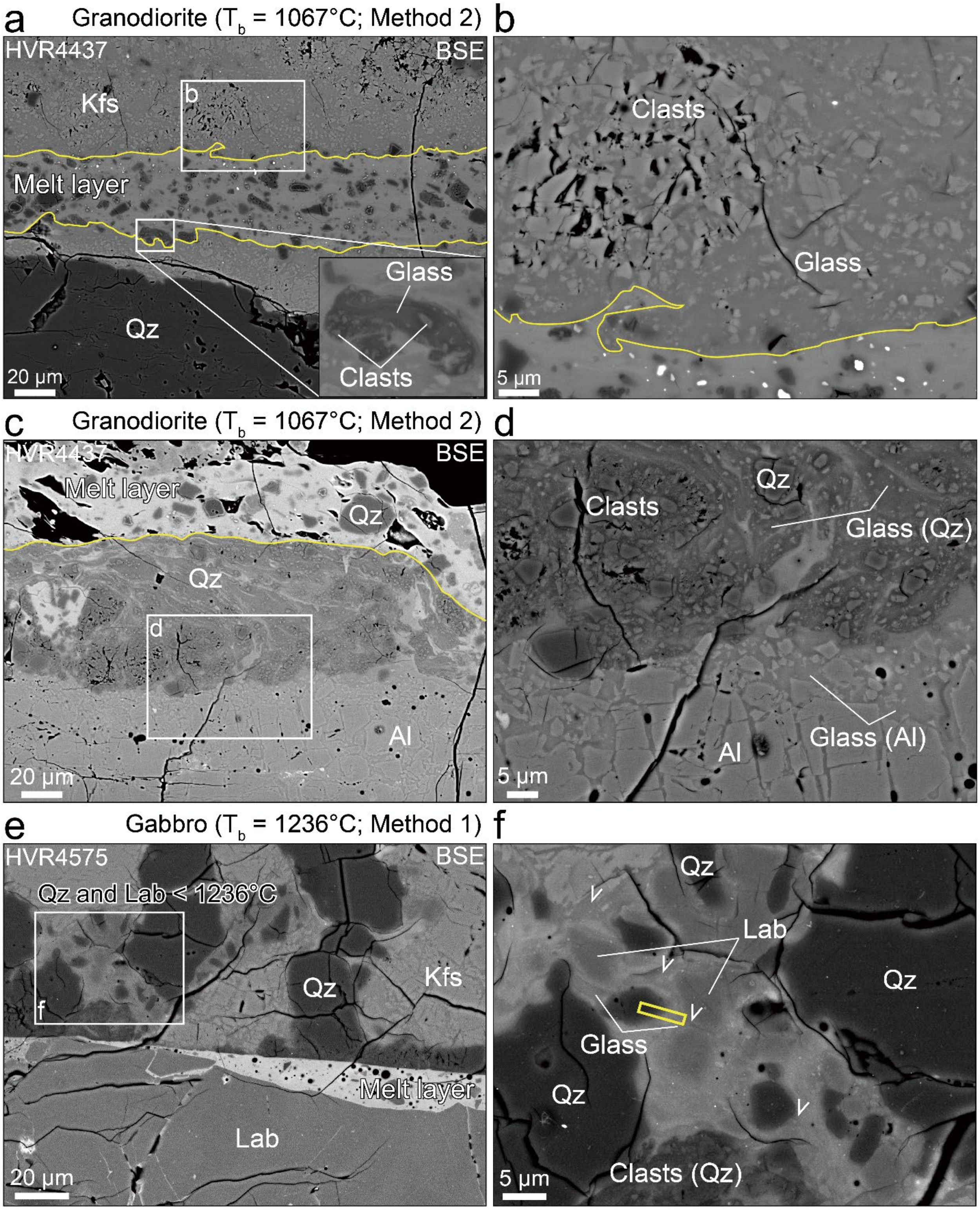

Fig. S9. BSE images showing melting along fractures and phase boundaries in host rocks. **a** Melting of highly fractured K-feldspar near the melt layer boundary. The melting temperature of K-feldspar in this area was lower than the melt layer boundary temperature (T_b). However,

due to the uncertainty of T_b (1067 °C) obtained by Method 2, it cannot be confirmed whether the melting of K-feldspar occurred at a lower temperature than its known T_m (1150 °C). The inset image shows a clast of solidified silica melt, indicating that the fractured quartz had possibly already melted. **b** Close-up view of the boxed area in **a** (the glass-rich area on the right may have evolved from a severely fractured area). **c, d** Melting of highly fractured quartz and albite. Note that the quartz, which is closer to the melt layer boundary than the albite, appears to be more extensively melted, and see also the glass network along the fractures in the albite. Considering the uncertainty of T_b (1067 °C), it is uncertain whether albite melting occurred at a lower temperature than its known T_m (1100 °C). However, it is highly likely from the silica glass that quartz melted at a much lower temperature than its known T_m (1720 °C). **e, f** Melting at the quartz/labradorite boundary. Glass is indicated by 'v' in **f**. See also fig. S10 for TEM observation results. The melting temperatures for quartz and labradorite in this area are < 1236 °C (T_b by Method 1), that is, they are below the known T_m for quartz (1720 °C) and labradorite (1344 °C); Qz, quartz; Al, albite; Ol, olivine; Kfs, K-feldspar; Lab, labradorite. BSE, back-scattered electron image.

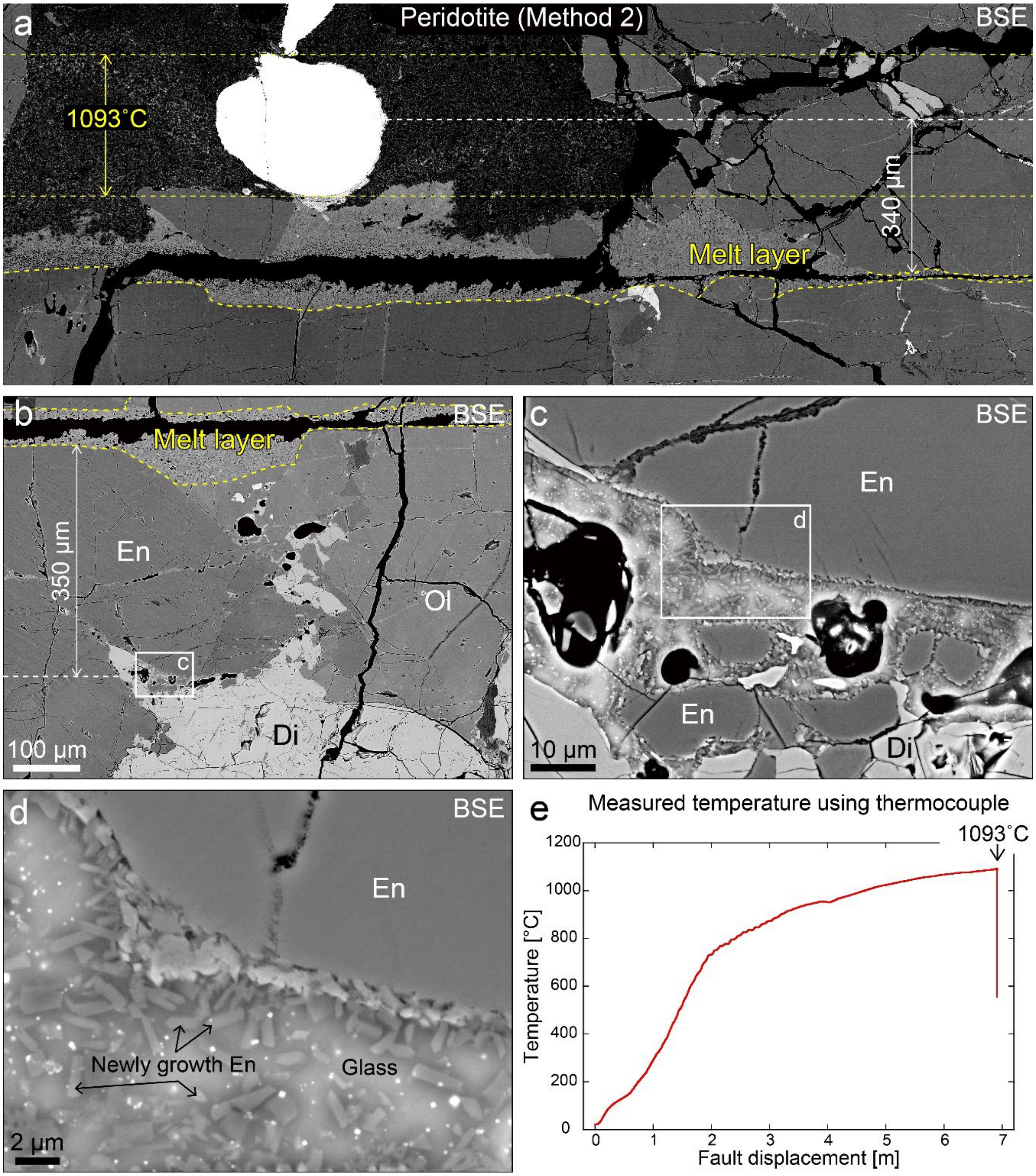

Fig. S10. BSE images showing melting along the phase boundaries in the wall rock and temperature measured by a thermocouple. a. Thermocouple tip located 340 µm away from the melt layer boundary. At the location, the measured temperature was 1093 °C**. b–d.** Microstructures of melting at the boundaries between enstatite (T_m = 1425 °C) and diopside (T_m = 1400 °C), located at 350 µm away from the melt layer boundary. Note the newly grown enstatite grains (arrows) and the glass. **e.** Temperature measured by the thermocouple shown in (a) indicates melting at the enstatite-diopside boundaries at 1093 °C or lower. En, enstatite; Di, diopside; Ol, olivine; BSE, back-scattered electron image.

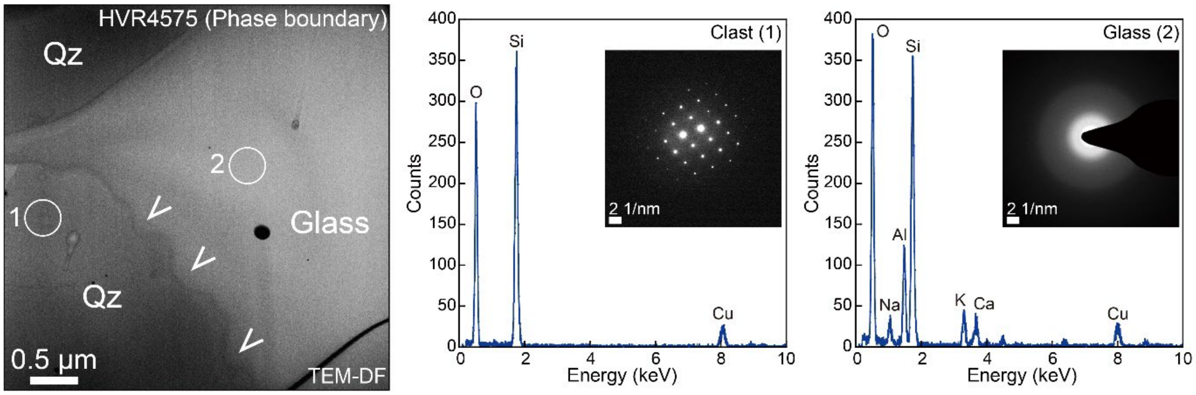

Fig. S11. TEM observation of the boundary area between quartz and labradorite (yellow-boxed area in Fig. S9f). The wavy boundary (marked by 'v') between β-quartz and glass indicates the melting of β-quartz (left TEM-DF image). The β-quartz (circle 1 in the left image) and glass (circle 2 in the left image) identities are confirmed by the diffraction patterns (central and right images). The SAED pattern and EDS data (right image) indicate that both labradorite and β-quartz were melted. Qz, quartz; TEM-DF, transmission electron microscope dark field image.

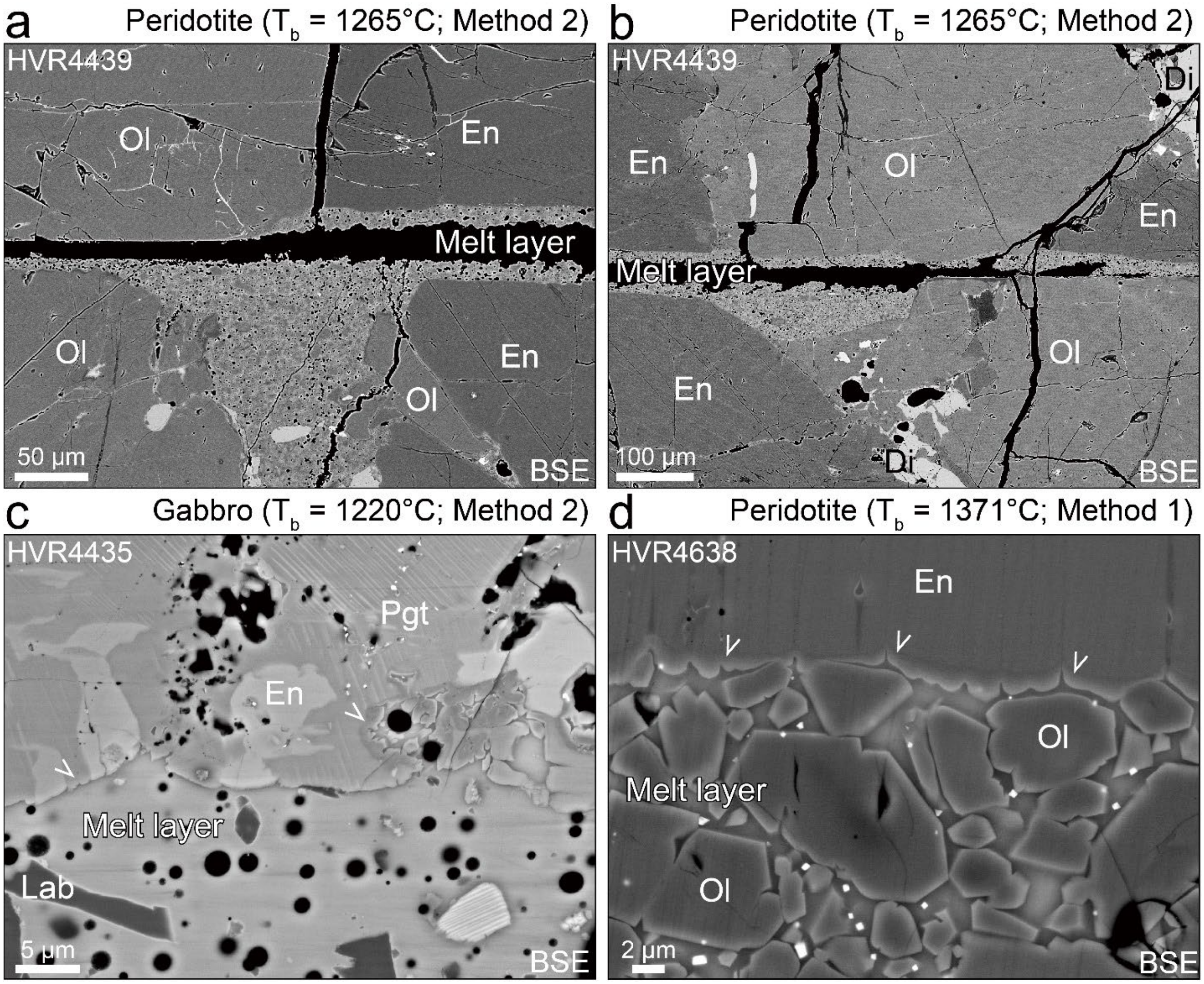

Fig. S12. BSE images showing melting along the host rock–melt (solid–liquid) boundary. **a, b** Olivine and enstatite melting along the melt layer boundary in peridotite. Melt layer boundary temperature, T_b by Method 2 = 1265 ℃. The injection of melt into the host rock in the center in **a** may have been caused by earlier dominant diopside melting. **c** Pigeonite melting in gabbro (marked by 'v'). T_b by Method 2 = 1220 ℃. **d** Enstatite melting in peridotite (marked by 'v'). T_b by Method 1 = 1371 ℃, which indicates enstatite melted at a lower temperature than its known melting point (1425 ℃). Ol, olivine; En, enstatite; Pgt, pigeonite; Lab, labradorite. BSE, back-scattered electron image.

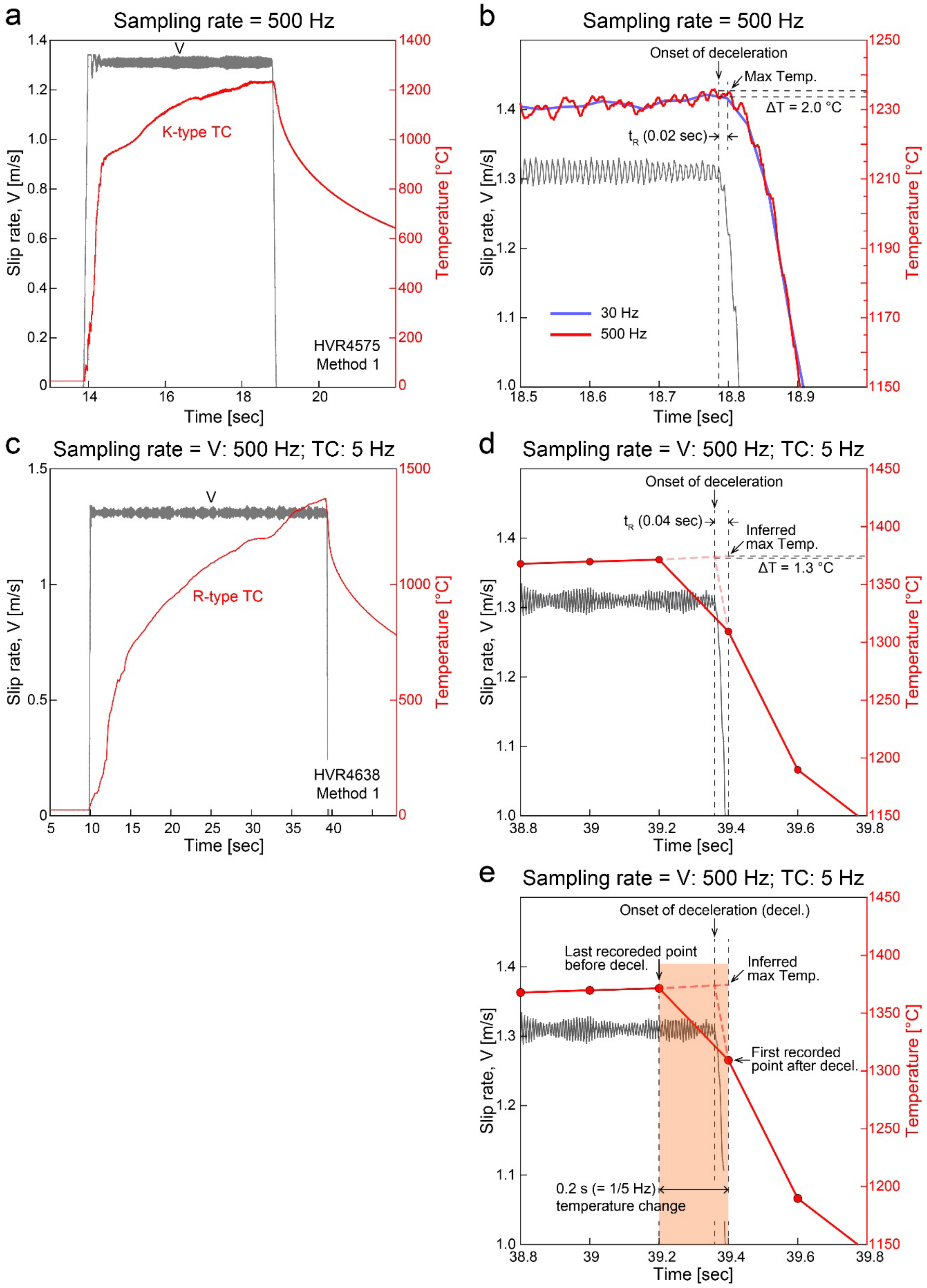
a
Sampling rate = 500 Hz
V
K-type TC
HVR4575
Method 1
Slip rate, V [m/s]
Temperature [°C]
Time [sec]
b
Sampling rate = 500 Hz
Onset of deceleration
Max Temp.
ΔT = 2.0 °C
t_R (0.02 sec)
30 Hz
500 Hz
c
Sampling rate = V: 500 Hz; TC: 5 Hz
R-type TC
HVR4638
Method 1
d
Sampling rate = V: 500 Hz; TC: 5 Hz
Onset of deceleration
t_R (0.04 sec)
Inferred
max Temp.
ΔT = 1.3 °C
e
Sampling rate = V: 500 Hz; TC: 5 Hz
Onset of deceleration (decel.)
Last recoreded point
before decel.
Inferred
max Temp.
First recorded
point after decel.
0.2 s (= 1/5 Hz)
temperature change

Fig. S13. Effect of data sampling rate and response time of thermocouples. a Temperature measured using a K-type TC during an experiment (HVR4575). **b** Close-up view of the temperature change at the final stage of the experiment. Original temperature data (red curve; sampling rate: 500 Hz) and data resampled at a rate of 30 Hz for the reduction of fluctuation (blue curve) are shown together. Since the TC tip is positioned at the boundary of the melt layer or inside the melt layer (Fig. S4), the temperature decrease should be recorded immediately after the slip deceleration indicating the onset of cooling if the TC response time is so short to be negligible. The upper limit of response time (t_R) of the TC in the experiment is 0.02 s, the time elapsed from the point of the onset of slip deceleration to the point at which the decreased temperature was first recorded. The underestimation of T_b due to the sampling rate is the difference between the highest measured temperature and the expected maximum temperature at the moment of the beginning of slip deceleration. The underestimation in the experiment is 1.8 °C. **c** Temperature measured using an R-type TC during an experiment (HVR4638). In the experiment, all the mechanical data except temperature were collected at a sampling rate of 500 Hz. Temperature data were sampled at a low rate of 5 Hz because the performance of the data logger for the R-type TC allowed only that level of the sampling rate. **d** Close-up view of the temperature change at the final stage of the experiment. In the experiment, t_R is 0.04 s. The underestimation of the maximum temperature due to the low sampling rate is <1.3 °C. **e** Determination of the response time (t_R) using the velocity (sampling rate, 500 Hz) and temperature data (sampling rate, 5 Hz). The temperature before the onset of the deceleration was slightly increasing, so it must have reached the maximum at the onset of deceleration and then began to decrease. The first recorded temperature after the onset of slip deceleration showed a reduced value. The time elapsed from the onset of slip deceleration to the first temperature recording was 0.04 s. Thus, the TC detected a decrease in temperature within 0.04 s, which may be an estimate of t_R.

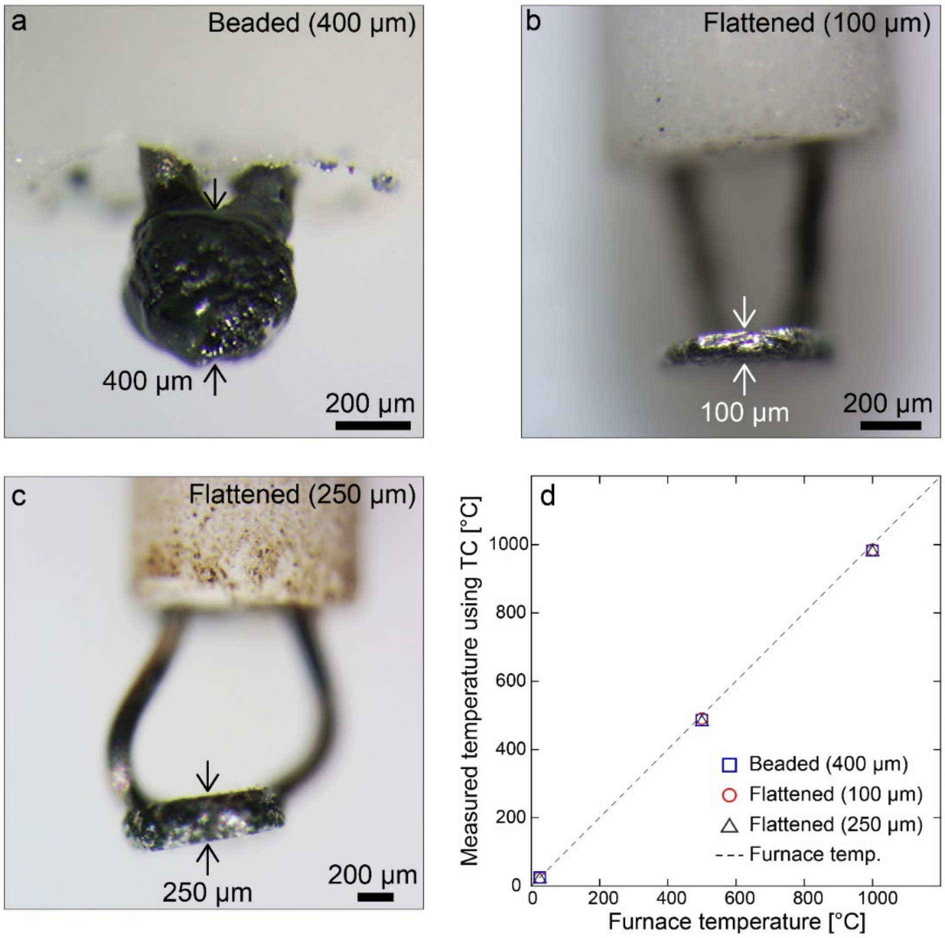

Fig. S14. Temperature measurement with different types of thermocouple (TC) tips. a A commonly used beaded-type TC tip. **b** A 100-μm-thick flattened TC tip. It was prepared by pressing a beaded-type tip. **c** A 250-μm-thick flattened TC tip. **d** Temperature measurement using the three types of TC tips at the furnace temperatures of 500 and 1,000°C. Note the nearly identical temperatures from the three kinds of TC tips (see also Table S7 for temperature data).

Table S1. Modal mineralogy of the host rocks used in the study.

Host rock	Mineral	Modal %
Granodiorite	Plagioclase (albite)	42.1
	Quartz	37.2
	K-feldspar	13.5
	Others (amphibole, mica and oxide minerals)	7.2
Gabbro	Plagioclase (labradorite)	63.3
	Pyroxene (enstatite and pigeonite)	19.4
	Quartz	5.7
	K-feldspar	5.1
	Others (amphibole, biotite and oxide minerals)	6.5
Anorthosite	Plagioclase (labradorite)	97.2
	Others (amphibole, pyroxene, quartz)	2.8
Peridotite	Olivine	73.5
	OPX (enstatite)	18.7
	CPX (diopside)	6.7
	Oxides	1.2

Table S2. Summary of friction experiments.

Host rock	Run No.	Normal stress (MPa)	Fault displacement (m)	Slip rate (m/s)	Peak Shear stress (MPa)	Steady state Shear stress (MPa)	Temperature measurement methods*
Granodiorite	HVR4437	15	5.89	1.3	11.10	3.08	K-TC
Granodiorite	HVR4441	15	7.34	1.3	9.44	4.97	K-TC
Granodiorite	HVR4637	15	1.30	1.3	8.30	2.68	K-TC
Gabbro	HVR4435	10	9.12	1.3	4.99	1.81	K-TC
Gabbro	HVR4575	10	6.44	1.3	6.38	2.13	K-TC
Gabbro	HVR4577	10	7.46	1.3	5.19	1.54	K-TC
Gabbro	HVR4615	10	7.03	1.3	2.47	1.77	K-TC
Gabbro	HVR4616	10	3.54	1.3	4.92	2.02	K-TC
Gabbro	HVR4626	10	14.33	1.3	4.94	1.52	K-TC
Gabbro	HVR4632	10	1.56	1.3	2.78	3.00	K-TC
Anorthosite	HVR4584	10	2.95	1.3	3.68	2.20	K-TC
Anorthosite	HVR4617	10	7.18	1.3	5.27	1.53	K-TC
Anorthosite	HVR4622-1	10	2.30	1.3	5.05	3.10	K-TC
Anorthosite	HVR4630-1	10	1.76	1.3	3.66	2.89	K-TC
Peridotite	HVR4439	13	6.91	1.3	5.49	2.08	K-TC
Peridotite	HVR4503	13	27.06	1.3	5.06	1.22	K-TC
Peridotite	HVR4581-1	13	30.97	1.3	4.66	1.26	R-TC
Peridotite	HVR4638	13	38.60	1.3	2.81	0.70	R-TC

*K-TC: K-type thermocouples; R-TC: R-type thermocouples

Table S3. Melt layer boundary temperatures (T_b).

	Method 1		Method 2	
	Run No.	Temperature	Run No.	Temperature
Acidic rock (Granodiorite)	HVR4637	1037℃	HVR4437	1067℃
			HVR4441	1077℃
Basic rock (Gabbro)	HVR4575	1236℃	HVR4435	1220℃
	HVR4616	1192℃	HVR4626	1295℃
Basic rock (Anorthosite)	HVR4630-1	1227℃	HVR4617	1315℃
	HVR4584	1209℃	HVR4622-1	1335℃
Ultrabasic rock (Peridotite)	HVR4581-1	1305℃	HVR4439	1265℃
	HVR4638	1371℃	HVR4503	1300℃
				1328℃

Table S4. Chemical compositions of olivine in the host rock and melt layer determined by electron microprobe analysis.

	SiO_2	TiO_2	Al_2O3	FeO	MnO	MgO	CaO	Na_2O	K_2O	Cr_2O_3	Total
Olivine in host rock (n = 3)	40.89	0.00	0.00	8.39	0.02	50.59	0.00	0.00	0.01	0.01	99.98
	5.97	0.00	0.00	1.02	0.01	11.02	0.00	0.00	0.00	0.00	18.03
Euhedral olivine in melt layer (n = 31)	41.86	0.01	0.30	6.94	0.11	51.08	0.44	0.03	0.00	0.22	101.00
	6.01	0.00	0.05	0.83	0.01	10.94	0.07	0.01	0.00	0.03	17.95
Clastic olivine in melt layer (n = 17)	41.30	0.01	0.12	8.37	0.14	50.32	0.22	0.01	0.01	0.05	100.56
	6.00	0.00	0.02	1.02	0.02	10.89	0.03	0.00	0.00	0.01	17.99

Table S5. Chemical compositions of the minerals in peridotite determined by electron microprobe analysis.

	Olivine		Diopside		Enstatite		Cr-spinel	
	mass%	atom%	mass%	atom%	mass%	atom%	mass%	atom%
SiO_2	40.89	5.97	52.71	7.62	55.71	7.63	0.06	0.01
TiO_2	0.00	0.00	0.40	0.04	0.07	0.01	0.01	0.00
Al_2O_3	0.00	0.00	4.51	0.77	3.89	0.63	57.61	10.68
FeO	8.39	1.02	1.92	0.23	5.78	0.66	11.55	1.52
MnO	0.08	0.01	0.08	0.01	0.03	0.00	0.10	0.01
MgO	50.59	11.02	16.09	3.47	34.34	7.01	20.16	4.73
CaO	0.02	0.00	23.08	3.58	0.39	0.06	0.01	0.00
Na_2O	0.00	0.00	1.04	0.29	0.04	0.01	0.02	0.01
K_2O	0.01	0.00	0.00	0.00	0.00	0.00	0.00	0.00
Cr_2O_3	0.01	0.00	0.50	0.06	0.30	0.03	9.08	1.13
Total	99.98	18.03	100.34	16.07	100.55	16.04	98.61	18.09

Table S6. Chemical composition of glass and newly crystallized olivine, and melting ratios of minerals.

	Glass and euhedral olivine grains (n = 10)	Case 1 : Total melting olivine (73.5%), diopside (6.7%), enstatite (18.7%), Cr-spinel (1.2%)	Case 2 : melting of olivine, diopside, and enstatite *Estimated best fitting ratio : olivine (53%), diopside (20%), enstatite (24.4%), Cr-spinel (2%)
	Average mass%	Average mass%	Average mass%
SiO_2	45.96	44.00	45.81
TiO_2	0.09	0.04	0.10
Al_2O3	3.11	1.72	3.00
FeO	6.16	7.51	6.47
MnO	0.15	0.07	0.07
MgO	38.17	44.92	38.81
CaO	4.70	1.64	4.72
Na_2O	0.25	0.08	0.22
K_2O	0.03	0.00	0.00
Cr_2O_3	0.31	0.20	0.36
Total	98.92	100.20	99.56

Table S7. Temperature measurement with different types of thermocouple tips.

Furnace temperature [°C]	Types of thermocouple tips		
	Beaded (400 μm) [°C]	Flattened (100 μm) [°C]	Flattened (250 μm) [°C]
25	25	25	25
500	487	491	485
1,000	983	985	983
